# Retinal Vessels Change in Primary Angle-Closure Glaucoma: The Handan Eye Study

**DOI:** 10.1038/srep09585

**Published:** 2015-04-30

**Authors:** Jianlu Gao, Yuanbo Liang, Fenghua Wang, Ran Shen, Tienyin Wong, Yi Peng, David S. Friedman, Ningli Wang

**Affiliations:** 1 Beijing Tongren Eye Center, Capital University of Medical Sciences, Beijing, China; 2 Liaocheng Clinical Hospital, Taishan Medical College, Shandong Province, China; 3 Handan Eye Hospital, Hebei Province, People's Republic of China; 4 Centre for Eye Research Australia, University of Melbourne, Melbourne, Australia; 5 Singapore Eye Research Institute, National University of Singapore, Singapore, Republic of Singapore; 6 Dana Center for Preventive Ophthalmology, Wilmer Eye Institute, Johns Hopkins University, Baltimore, Maryland; 7 Department of International Health, Bloomberg School of Public Health, Johns Hopkins University, Baltimore, Maryland

## Abstract

Glaucoma is the second leading cause of blindness worldwide. To examine the relationship between angle closure and the retinal vessel diameter in Chinese adults, we conducted Handan Eye Study (HES), a large population-based cross-sectional study, which enrolled 6830 participants >30 year-old living in 13 randomly selected villages of Yongnian County. After adjusting for age, gender, spherical equivalent (SE), diabetes, and hypertension, the mean central retinal artery equivalent (CRAE, μm) was 127.1 ± 7.0 and 145.6 ± 4.4 in primary open-angle glaucoma (POAG) and primary angle closure glaucoma (PACG), respectively; narrower than that in normal control (156.1 ± 0.4), primary angle-closure suspect (PACS) (156.3 ± 1.1) or primary angle closure (PAC) (156.0 ± 3.4) (*P* = 0.001). The mean central retinal vein equivalent (CRVE, μm) was 229.0 ± 5.9 and 215.8 ± 9.5 in POAG and PACG, respectively; narrower than that in normal control (238.3 ± 0.5), PACS (241.2 ± 1.4) or PAC (242.2 ± 4.6) (*P* = 0.001). There was no significant difference in the mean CRAE or CRVE between PACG and POAG. Compared to the normal control (0.66), the mean arterio-venous ratio (AVR) was smaller in POAG (0.64) and PACG (0.59), whereas larger in PACS (0.65) and PAC (0.67) (*P* = 0.003). To conclude, PACG and POAG individuals have narrower retinal arteries and veins.

Glaucoma is a critical public health problem and the second (after cataracts) leading cause of blindness worldwide. Primary open-angle glaucoma (POAG) is the major reason for incurable visual impairment^1^. It is characterized by progressive loss of retinal ganglion cells, resulting in glaucomatous optic neuropathy (GON). The etiology of POAG is not well established. Potential risk factors include intraocular pressure (IOP), age^2^, race, family history, genetic^3^, myopia^4^, intraocular pressure^5^, and poor perfusion of optic nerve head or ganglion cell layer^6^,^7^,^8^,^9^. The common consequence for potential etiologies is optic nerve head damage, which is secondary to primarily ganglion cell axon loss; however, loss of blood vessels and glial cells has also been observed. There are many postulated mechanisms of ganglion cell damage. It has been reported that eyes with glaucomatous damage had narrower retinal arteriolar diameters (183 ± 2.6 μm) than normal eyes (194 ± 0.4 μm) or ocular hypertension (195 ± 1.6 μm)^8^. A prospective cross-sectional study observed significant reduction in the speed and flow of retinal blood in POAG patients compared to normal controls. It is unclear whether an ischemic process can lead to GON or whether the narrowing of retinal blood vessel is secondary to glaucoma^10^.

In primary angle closure (PAC), an eye has a primary anatomic narrow angle and trabecular obstruction in the peripheral iris, caused by peripheral anterior synechiae (PAS), elevated IOP, iris whorling or sectoral atrophy, and excessive pigment deposition on the trabecular surface. The eye does not have glaucomatous damage of the optic nerve.

In primary angle-closure suspect (PACS) or anatomic narrow angle, the anterior chamber angle recess has an abnormally narrow angular width. The peripheral iris is located close to, but not touching, the posterior pigmented trabecular meshwork (TM). No PAS are present. IOP, optic nerve, and visual field are normal.

Primary angle closure glaucoma (PACG) is diagnosed when iridotrabecular contact is present in three or more quadrants of the drainage angle in the presence of documented optic nerve damage and visual field loss. It has been proposed that the GON in PACG is caused by high IOP and reduced circulation^5^,^11^. We hypothesize that the retinal vessel diameter of PACG patients may confer the changes in circulation and correlate with the subsequent GON. In China, 1.0–1.5% of people ≥40 year-old have PACG^6^,^7^,^8^,^9^, which is much higher than that in Caucasian (0.1%–0.6%)^12^,^13^. This high prevalence has enabled the Handan Eye Study (HES), a large population-based study on eye diseases in northern China, obtained a larger group of PACG patients. Here, we analyzed the differences in retinal vessel diameters among PACS, PAC, PACG, POAG and normal participants, after adjusting for age, gender, refraction, and other possible confounders.

## Methods

### Study population and design

The HES is a population-based study on vision, common eye diseases, and other outcomes of health problems. The study was conducted according to the Declaration of Helsinki, and was approved by Ethical Committee of Beijing Tongren Hospital. Written informed consent was obtained from each of the participants. Details of the study design, sampling plan, and baseline data have been described previously^14^,^15^. In brief, 7557 eligible individuals older than 30 years living in Yongnian County, Handan City, Hebei Province, China, were identified from 13 randomly selected villages using a stratified, clustered, and multi-staged sampling technique, with probabilities proportionate to the size of the population in each cluster. Among the population in Yongnian County, 90% are farmers, and 98% are Han Chinese.

All eligible subjects were invited to visit Yongnian County Hospital for a serial examination. Those who declined to visit the hospital were offered a simplified evaluation at a temporary field site established in the village; those who declined to visit the temporary site were offered a limited examination conducted at home. All fieldwork was conducted from October 2006 to October 2007. Finally, 6830 subjects completed the examination, and 6656 of 6830 (97.45%) participates with detailed demographic and clinical information were included in final data analysis.

### Procedures and definitions

For the baseline evaluation, 5909 of 6830 participants were examined in the county hospital, 807 in a temporary study site at the village, and 114 at home. At the county hospital and the village temporary site, fundus photographs of two fields (optic disc and macula) were taken from both eyes of each participant after pupil dilation using a digital retinal camera. The photographs were taken by Canon CR-DGi with a 20D SLR back (Canon, Japan). Intraocular pressure (IOP) was measured with a Kowa applanation tonometer HA-2 (Kowa Company Ltd. Tokyo, Japan). Visual field test was conducted using the Humphrey Visual Field Analyzer 750i (Carl Zeiss, Jena, Germany) in all participants who were suspected to have glaucoma. Lens thickness and axial measurements were obtained using an A-mode ultrasound device. Other details of the eye examinations have been described previously, including the baseline visual acuity (VA), and the spherical equivalent (SE) measured using subjective refraction^7^,^14^.

**Figure 1:**
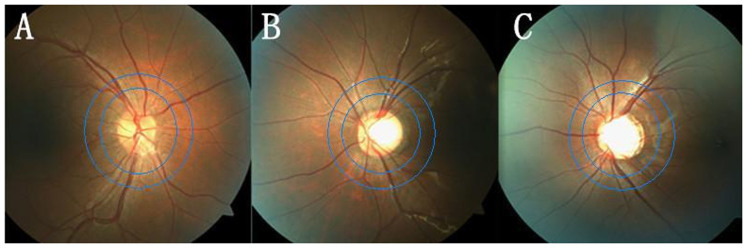
The fundus photographs of Control (A), PACG (B) and POAG (C), and the measured area (between2 blue circles 0.5 to 1.0-disc diameter away from the optic disc margin) for retinal arteriole and vein diameter.

The retinal vessel diameter measurement was performed as previously described^8^,^16^. Graders were trained at the Retinal Vascular Imaging Centre at The University of Melbourne prior to the assessment of the photographs. Retinal vessel diameters from digital retinal images were measured using a computer-based program by trained graders who were blinded to the status of each of the participant. For an image, all arterioles and venules coursing through an area of one-half to one-disc diameter away from the optic disc margin were measured and summarized as the central retinal artery equivalent (CRAE) and central retinal vein equivalent (CRVE), respectively. These equivalents represent the average caliber of arterioles and venules of the eye (Figure 1).

The limbal anterior chamber depth (LACD) was measured by slit lamp examination. Gonioscopy was performed in one out of every ten participants as well as in those with LACD ≤40% corneal thickness using Goldmann Magnaview lens (Ocular Instruments, Bellevue, Washington, USA) at 25× magnification under low ambient illumination by an experienced ophthalmologist. A narrow vertical beam of 1 mm in length was offset vertically for superior and inferior quadrants, and horizontally for nasal and temporal quadrants. Small movements of the lens were allowed to visualize the drainage angle, but large movements were avoided because of the possibility of indentation. Dynamic examinations with Goldmann lens were performed after the static gonioscopy of the four quadrants was completed. If a satisfactory examination could not be achieved with Goldmann lens, 4-mirror Sussman lens (Ocular Instruments, Bellevue, Washington, USA) were used. The Spaeth Gonioscopic Grading System was used to record the results^17^.

The vertical cup-to-disc ratio (VCDR) was calculated to exclude the presence of peripapillary atrophy and the scleral ring of Elschnig. The margin of the cup was defined by stereoscopic view as the point of the maximum inflection of the vessels crossing the neuroretinal rim. Standard photographs for VCDR from 0.1 to 1.0 in 0.1 increment were used for the grading process.

Every 10th participate was systematically sampled with a visual field test using 24-2 Swedish Interactive Threshold Algorithm (SITA) fast program. In addition, all participants with angle closure disease or suspected glaucoma had SITA standard visual field test. Tests were repeated twenty minutes later if the glaucoma hemifield test (GHT) was outside the normal limits, on the borderline, or if the test was unreliable (i.e., fixation loses >20%, false positives >33%, or false negatives >33%).

GON was diagnosed based on the consensus opinion of three glaucoma specialists after review of all relevant information including patient history, VA, and optic nerve stereo photographs. POAG was diagnosed if 1) GON was present without identifiable secondary causes; and 2) angle closure was excluded on gonioscopy^18^. PACS was diagnosed if the posterior trabecular meshwork was not visible for 180° or more on gonioscopy. PAC was defined as PACS with IOP ≥21 mmHg or the presence of peripheral anterior synechiae (PAS) without GON. PACG was defined as PAC with GON^13^. The eyes without PACS, PAC, PACG, POAG, or non-glaucomatous neuropathy were defined as the normal.

Certified nurses measured height, weight, pulse rate, and blood pressure according to standardized protocols. Total cholesterol, total triglycerides, low density lipoprotein (LDL) cholesterol, high density lipoprotein (HDL) cholesterol, and glucose level in fasting blood were analyzed using certified protocols. Diabetes mellitus (DM) and hypertension were defined as previously reported^14^. Migraine and angina were diagnosed using interviewer-administrated questionnaires.

### Statistics

Patients diagnosed with ocular hypertension without GON were assigned to the “normal” group(control), and patients diagnosed with normal-tension glaucoma were assigned to the POAG group, because previous studies have found no difference in retinal vessel diameter among these groups^8^.

All statistical analysis was performed using SAS software^9^,^13^. Data from the right eyes of all participants in the normal controls and binocular glaucoma participants was analyzed. Data from the glaucomatous eye in participants with monocular glaucoma was analyzed. The baseline data was analyzed using covariance, adjusted by potentially confounding variables including age, gender, intraocular pressure (IOP), spherical equivalent refractive error (SE), diabetes (DM), and hypertension in logistic regression models. ANCOVA was used to adjust the influences of age, gender, IOP, SE, DM, and hypertension on retinal vessel diameter or its measurement. A *P* value <0.05 was considered as statistically significant.

## Results

**Figure 2:**
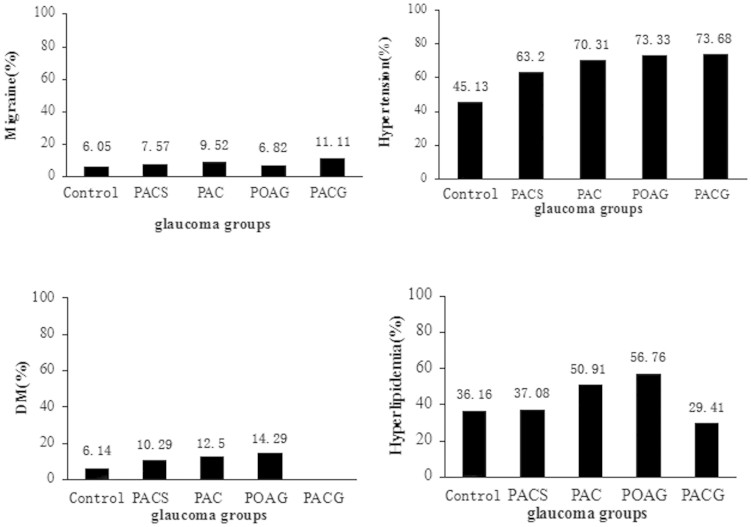
Percent of Migraine, Hypertension, DM, and Hyperlipidemia in Control(Non-glaucoma)/PACs/PAC/POAG/PACG group.

A total of 6716 participants completed the ocular examination. Sixty participants were excluded because the fundus images were non-gradable. A total of 6656 participants (97.45%) were included in data analysis(3088male,3568female). Angle closure was diagnosed if the posterior pigmented trabecular meshwork was invisible for ≥180°. Primary angle-closure suspect (PACS) was diagnosed if iris trabecular contact ≥180 degree with intraocular pressure (IOP) <21 mmHg, without peripheral anterior synechiae nor glaucomatous optic nerve damage. Primary angle closure (PAC) was diagnosed if angle closure presented with IOP ≥21 mmHg and/or peripheral anterior synechiae, without glaucomatous optic neuropathy. Primary angle closure glaucoma (PACG) was diagnosed if angle closure and glaucomatous nerve damage presented. Average retinal vessel diameters were summarized as arteriolar and venular equivalents. Among them, 731 patients were diagnosed as PACS(), 64 as PAC, 19 as PACG, and 54 as POAG. The baseline characteristics of all of the participants were shown in Table 1. Participants with GON were older and had a higher prevalence of DM and hypertension. The IOP was increased in PACG, PAC and POAG groups. The VCDR in PACG (0.85) and POAG (0.66) groups were larger than that in the normal control (0.41), PACS (0.42) and PAC (0.40) groups. The participants with PACS and PAC were more likely to have hyperopia, and those with POAG had a higher prevalence of myopic and hyperlipidemia. After adjusting for age and gender, there were significant differences in SE, IOP, DM, and hypertension among different groups (Figure 2).

**Figure 3:**
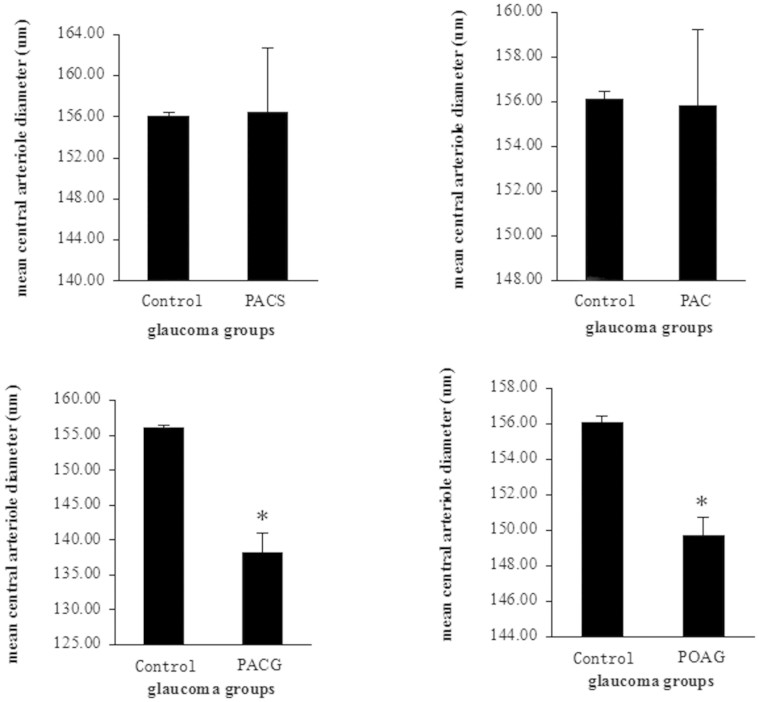
The retinal arteriole diameters (CRVE) compared to the Control(Non-glaucoma) group, *p < 0.05.

Figure 3 summarized the retinal arteriole diameters in the normal and glaucomatous groups after adjusting for age, gender, SE, DM, and hypertension. The mean CRAE was significantly narrower in the PACG (127.1 ± 7.0 μm) and POAG (145.5 ± 4.4 μm) groups than that in the normal control (156.1 ± 0.4 μm), PACS (156.3 ± 1.1 μm), and PAC (156.0 ± 3.4 μm) groups (*P* = 0.001). However, the extent of narrowing in CRAE was comparable between PACG and POAG group (95% CI: −1.5 μm to 35.6 μm).

**Figure 4:**
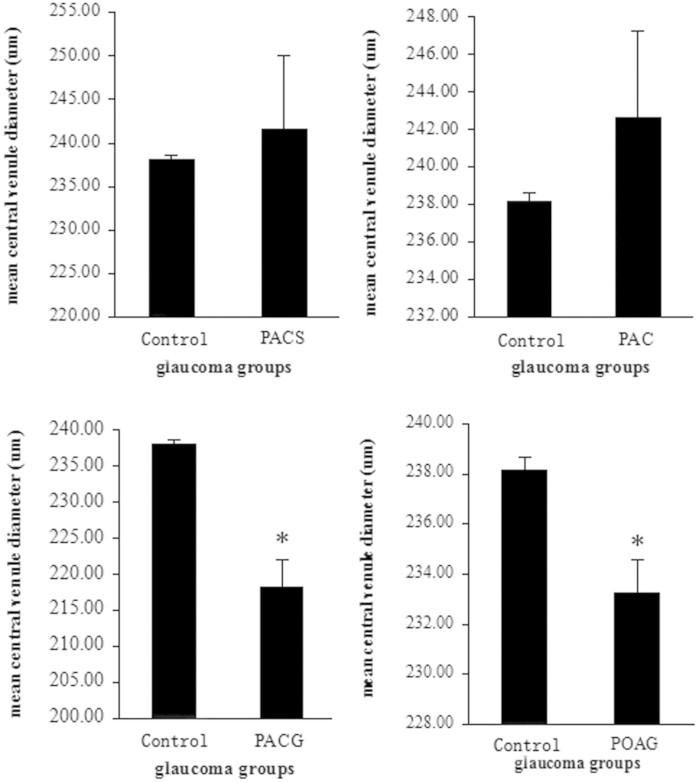
The retinal vein diameters (CRVE) compared to the Control(Non-glaucoma) group, *p < 0.05.

As shown in Figure 4, the mean CRVE was significantly narrower in the PACG (215.8 ± 9.5 μm) and POAG (229.0 ± 5.9 μm) than that in the normal control (238.3 ± 0.5 μm), PACS (241.2 ± 1.4 μm), and PAC (242.2 ± 4.6 μm) (*P* = 0.012). However, the extent of narrowing in CRVE was comparable between PACG and POAG (95% CI: −14.2 μm to 35.8 μm).

**Figure 5:**
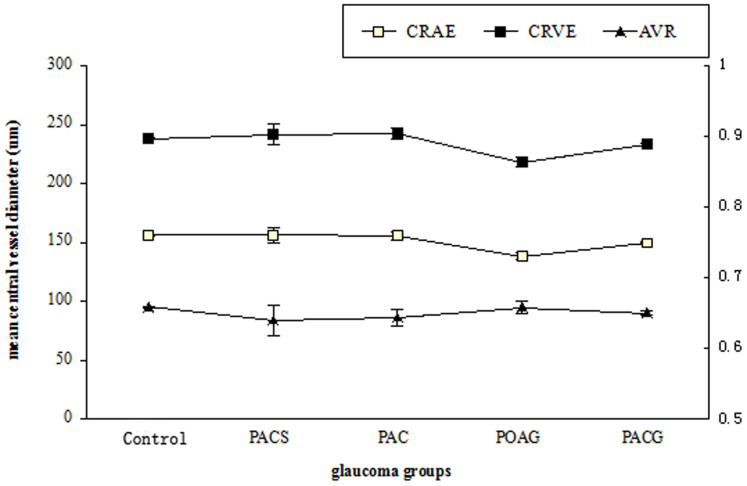
The retinal arteriole/vein diameter (CRAE/CRVE) and arteriole/vein ratio (AVR) in Control(Non-glaucoma) and glaucoma groups. *P* = 0.003 compared between PACG and Control(Non-glaucoma) groups.

The mean AVR was shown in Figure 5. After adjusting for age, gender, SE, DM, and hypertension, the AVR in PACG (0.59) group was smaller than that in the normal control (0.66) (*P* = 0.003). There was no significant change in the AVR from PACS (0.65), PAC (0.67) and POAG (0.64) groups compared to the normal control.

## Discussion

In this HES, retinal blood vessel diameter was measured by the method used in Blue Mountains Eye Study (BMES) and Beaver Dam Eye Study (BDES)^8^,^16^. The influence of age, gender, IOP, SE, DM, and hypertension on the vessel diameter or its measurement was adjusted by ANCOVA. We found that the diameters of retinal artery and retinal vein in POAG were significantly narrower than those in the normal control (*P* = 0.001), with a reduction of 10.6 μm (6.8%) and 9.3 μm (3.9%), respectively. This result was consistent with those reported by BMES and BDES^8^,^16^, and another Chinese population-based study named Beijing Eye Study (BES), where the retinal artery was narrower in the glaucomatous eyes^19^. The BES did not use the same method to measure retinal vessel diameter and did not separately analyze the data from the PACG participates. In our HES population, 19 participants with PACG, 64 with PAC and 731 with PACS had data of retinal vessel diameters. The relatively more PACG individuals in our study may result from the large sample size of HES and higher prevalence of PACG (as high as 1.0% among people aged ≥40 year-old) in Chinese population^20^. After adjusted the influence of age, gender, IOP, SE, DM, and hypertension, the diameters of the retinal artery and retinal vein in PACG were smaller than those in normal control, with a reduction of 29.0 μm (18.6%) and 22.5 μm (9.4%), respectively. So our data showed that retinal vessel narrowing exists in not only POAG, but also in other kind of glaucomatous eyes such as PACG. To our knowledge, very few studies on retinal vessel diameter in PACG had been reported before HES, especially such a large population-based study. However, our data did not provide evidence that angle closure process is related to the change of retinal vessel diameter.

Previous hospital-based and population-based studies conducted in Europeans and Africans reported the narrowing of peripapillary retinal blood vessels in patients with glaucoma^8^,^21^,^22^. The blood circulation contributes to the development and progression of GON in POAG patients. However, the Rotterdam Study, which was a population-based prospective cohort study^23^, analyzed the baseline retinal vessel diameters of 5517 participants and found no significant difference between non-glaucoma participants and 74 POAG individuals after a mean follow-up time of 6.5 years. Their results may indicate that blood vessel narrowing in glaucoma is part of the disease process instead of a cause of optic nerve damage. In our HES, the CRAE and CRVE were narrower in PACG and POAG than those in normal control, PAC or PACS. This finding further supports the notion that the narrowing of retinal vessels results from the glaucoma process.

The elevated IOP and structure changes of optic cup in eyes of glaucoma were considered to be related to the retinal vein obstruction. A retrospective study found that 18 of 83 patients initially presenting with central retinal vein occlusion had been diagnosed with POAG^25^. A hospital-based study found that central retinal vein collapse pressure was significantly higher in POAG (26.1 relative unit) than that in age-matched controls (6.1 relative unit)^24^. The obstruction might be manifested by the enlargement of the retinal vein diameter. To the contrary, BMES did not show an enlarged retinal vein or decreased AVR in POAG^8^. In our study, the retinal vein diameter in POAG and PACG was narrower than that in normal control. The AVR in the POAG was comparable to that in normal control, PACS, and PAC. Thus, BMES and HES may not support the previous notion that a higher retinal vein collapse pressure presents in the glaucomatous eyes.

In the HES, the mean CRAE of non-glaucomatous subjects was 156.09 μm, which is significantly narrower than that of the BMES (194.04 μm). The multivariate-adjusted CRVE in non-glaucoma was 238.25 μm, which is larger than that in the BMES (225.58 μm). The AVR in HES (0.66) was smaller than that in BMES (0.86) or BDES (0.69)^26^. Our data was similar to a study conducted in Asian Malay population aged 40–80 year-old^27^, with an AVR of 0.64 in normal control and 0.65 in glaucoma. A large vein diameter can be caused by genetic factors or an increased venous pressure; and the latter may correlate with the pathogenesis of PACG and other eye diseases. Whether the difference in AVR or the retinal arterial/vein blood pressure are related to ethnicity needs further investigation.

A retinal vessel with white sheath was detected in a PACG patient in the HES. This may cause a potential difficulty in setting-up vascular boundaries and thus introduce inaccuracy in the measurements of retinal vessel diameters. Among 6716 participants, the retinal vessel was non-gradable in a PACG due to the whit sheath of the retinal vessel. To reduce the bias between different groups, graders were trained at the Retinal Vascular Imaging Center at the University of Melbourne.

In conclusion, using the same computer-assistant method as the BMES and the BDES, we found that PACG and POAG patients had significantly narrower retinal vessel diameters than those of the normal control, PACS and PAC, after adjusting for age, gender, IOP, SE, DM, and hypertension. The AVR in our population was similar to that of Asia Malay ethnicity but smaller than that in the Caucasians. We propose that the retinal vessel narrowing in glaucoma is secondary to the loss of retinal ganglion cells.

## Author Contributions

J.G., Y.L. and N.W. wrote the main manuscript text. R.S., T.W. and F.W. analyzed fundus and retinal vessel diameter. N.W. and D.F. contributed to the diagnosis of glaucoma. Y.P. analyzed data and prepared table and figures. All authors reviewed the manuscript.

## Figures and Tables

**Table 1:** Characteristics of participants for retinal vessel examination in the Handan Eye study

	Normal (n = 5788)	PACS (n = 731)	PAC (n = 64)	POAG (n = 54)	PACG (n = 19)	P*
Continuous variables (mean ± SD)						
Age (years)	50.8 ± 11.8	59.7 ± 9.0	61.3 ± 8.9	61.2 ± 10.4	64.0 ± 13.9	<0.001
BMI	24.5 ± 3.8	24.4 ± 3.7	25.2 ± 4.0	23.6 ± 3.1	23.9 ± 4.3	0.276
SE (D)	−0.22 ± 1.79	0.64 ± 1.28	0.52 ± 2.14	−0.68 ± 2.59	0.08 ± 1.48	<0.001
IOP (mm Hg)	15.0 ± 2.8	14.8 ± 2.8	19.0 ± 5.0	16.9 ± 3.11	24.3 ± 13.4	<0.001
Pulse (times/min)	77.4 ± 2.0	78.9 ± 12.2	81.2 ± 14.0	80.2 ± 13.4	77.4 ± 13.9	0.002
MBP (mm Hg)	97.4 ± 14.2	100.4 ± 14.0	103.4 ± 13.7	102.5 ± 16.3	100.3 ± 13.1	<0.001
CRAE (μm)	154.9 ± 26.6	155.4 ± 20.2	159.0 ± 38.7	143.7 ± 20.5	128.6 ± 35.8	<0.001
CRVE (μm)	235.9 ± 36.2	240.7 ± 30.0	241.1 ± 41.0	223.9 ± 33.6	231.3 ± 57.4	<0.001
AVR	0.66 ± 0.09	0.65 ± 0.09	0.68 ± 0.23	0.65 ± 0.11	0.59 ± 0.22	<0.001
VCDR	0.41 ± 0.11	0.42 ± 0.10	0.40 ± 0.10	0.66 ± 0.18	0.85 ± 0.18	<0.001
Categorical variables (%)						
Migraine	6.05	7.57	9.52	6.82	11.11	0.214
Hypertension	45.13	63.20	70.31	73.33	73.68	<0.001
DM	6.14	10.29	12.50	14.29	0.00	<0.001
Hyperlipidemia	36.16	37.08	50.91	56.76	29.41	0.016

Normal eye: eyes of participants without glaucoma (PACS, PAC, PACG, POAG) or non-glaucomatous neuropathy; BMI: body mass index; SE (D): spherical equivalent (diameter); IOP: intraocular pressure; MBP: mean arterial blood pressure; DM: diabetes mellitus; CRAE: central retinal artery equivalent; CRVE: central retinal vein equivalent; AVR: ratio of CRAE/CRVE. VCDR: vertical cup disc ration. VCDR: vertical cup-to-disc ratio. *P value was from chi-square test (2-sided).
